# TransmiRisk-AI: an agentic AI-based risk assessment framework for zoonotic diseases using integrated surveillance data

**DOI:** 10.3389/fdgth.2026.1915858

**Published:** 2026-09-30

**Authors:** Ramya Jeyachandran, Rama Parvathy Lakshmanan

**Affiliations:** 1 School of Electronics Engineering, Vellore Institute of Technology, Chennai, India; 2 School of Computer Science and Engineering, Vellore Institute of Technology, Chennai, India

**Keywords:** agentic AI, autonomous monitoring systems, decision support, multi-agent systems, one health, real-time outbreak monitoring, zoonotic surveillance

## Abstract

**Introduction:**

Zoonotic disease outbreaks are increasing in frequency and complexity, highlighting the need for intelligent surveillance systems capable of real-time monitoring and risk assessment. Existing surveillance approaches are often reactive, fragmented, and dependent on manual analysis, limiting timely public health responses.

**Methods:**

This study presents TransmiRisk-AI, an agentic AI-driven surveillance framework designed to support automated zoonotic disease risk monitoring and decision-making. The framework integrates a hybrid risk scoring model with a multi-agent architecture comprising data ingestion, risk assessment, alert generation, execution, logging, and orchestration agents. These agents collaboratively automate data collection, preprocessing, risk evaluation, outbreak monitoring, and alert dissemination. To enhance situational awareness, the system incorporates outbreak intelligence from WHO Disease Outbreak News and dynamically updates disease risk profiles based on outbreak recency, frequency, and severity. An interactive Streamlit dashboard provides transparent visualization of risk categories, outbreak trends, and system activities.

**Results:**

Case studies involving the Nipah virus and Crimean-Congo hemorrhagic fever demonstrate the framework's ability to adapt to evolving epidemiological conditions.

**Discussion:**

TransmiRisk-AI offers a scalable and interpretable digital health solution that supports proactive surveillance and timely decision-making within a One Health context.

## 1 Introduction

The issue of zoonotic diseases is an acute and dynamic challenge to the health of people worldwide, caused by a complicated relationship among human, animal, and environmental systems (1). The growing number of emerging infectious diseases, such as Ebola virus disease, Nipah virus infection, and avian influenza, underscores the importance of having surveillance systems that can quickly identify, evaluate, and counter dynamic transmission threats (2). Traditional surveillance methods, using manual reporting and retrospective analysis, tend to be reactive in nature and are unable to deliver timely and data-driven insights to proactively intervene (3).

In recent years, artificial intelligence (AI) has made it possible to create intelligent surveillance systems with the ability to handle large and heterogeneous volumes of data, such as outbreak reports, environmental data, and epidemiological records (4). Examples of AI-powered tools (event-based surveillance systems, digital disease detection tools) have shown that they can help detect the initial signs of an outbreak and enhance situational awareness. Nevertheless, most of these systems are still constrained by disaggregated data integration, lack of automation, and inability to provide continuous risk assessment and prioritization of various diseases.

The major weakness of the current AI-based surveillance systems lies in the fact that they are based on fixed or disease-specific models that fail to dynamically change in response to changing outbreak conditions. Also, the majority of systems are labor-intensive to process and analyze data and make decisions, which makes them less efficient and scalable in practice in the context of public health. The lack of synchronized working procedures and automated pipelines also limit the possibility to convert raw material into an actionable intelligence in real time (5).

A promising next-generation form of surveillance is agentic artificial intelligence, where autonomous, goal-oriented systems, sensitive to their environment, perform tasks using coordinated activities. Through the use of multi-agent architectures, agentic AI systems can break down complex workflows into specialized functions including data ingestion, risk computation, alert generation and decision support. Such systems allow uninterrupted surveillance, adaptive learning and automatic response systems, which increases efficiency and responsiveness of surveillance systems (6).

Herein, this paper suggests TransmiRisk-AI, an agentic AI-based autonomous surveillance system to monitor the risk of zoonotic diseases in real-time. The suggested system will combine a hybrid risk assessment model with a multi-agent structure comprising data ingestion, risk evaluation, alerting, execution, logging, and orchestration agents. The framework facilitates ongoing data integration, real time recalculation of risks based on real time outbreak intelligence, and automatic alerting of high-risk diseases. An interactive dash-board is created to offer a clear visualisation of the risk categories, outbreak trends, and system operations.

The concept of agentic AI is autonomous systems that have the ability to perceive their surroundings and perform actions to meet desired objectives and the form of intelligence that replicates human cognitive processes, including learning and solving problems (7). This idea has become popular because AI technologies, such as intelligent agents, now form part of everyday life, which makes people discuss the legal and moral consequences of AI technologies (8). The development of agentic AI is highly associated with the development of interactive systems especially in the virtual worlds where the agentic AIs can develop and perform in complex situations (9). Moreover, the human-machine relations are becoming more interdependent, with AI systems being created to benefit the decision-making process in many applications. Therefore, agentic AI is an important boundary in the history of technology as well as the history of ethics.

There are a number of advantages to taking an agentic approach to artificial intelligence systems, especially in the operational areas of improving autonomy, decision-making, and user trust. Using the natural ability to adapt and optimize within a dynamic environment, AI as agents can perform independent actions to adapt through reinforcement learning which is apparent in multi-agent systems in applications of the IoT (10, 11). This enables proactive behavior and therefore the systems can identify any pattern and assume the needs of the user, which enhances user experience and satisfaction. Moreover, explainable AI (XAI) can be integrated, thereby establishing transparency, enabling users to know the reason AI made this decision, which is essential in establishing trust (12). Moreover, conceptual frameworks describing the idea of artificial agency may facilitate the connection between theory and practice, thus maximizing the usefulness of AI in research and practice settings (13). In general, an agentic approach does not only make the AI systems more functional, but also closer to human-like processes of decision making (14).

Now that agentic artificial intelligence systems have the ability to catalog and predict zoonotic disease outbreaks earlier, the source of data available to use to detect and forecast these outbreaks is immense through the use of the large volumes of open-source data and highly developed machine learning algorithms. Such systems, including EPIWATCH and BlueDot, employ artificial algorithms to examine various pieces of information, including social media, news stories, and health records, and therefore spot the possible early signs of an outbreak before conventional health authorities do (15, 16). As an example, EPIWATCH was able to predict the first signs of Mpox outbreaks by tracking reports of rash and fever cases, which proves its ability to issue the necessary warnings in time (16). Moreover, it is possible to use AI models to synthesize complex epidemiological data, which makes them useful in timely responding and making informed decisions by the officials of the health sector, thereby reducing the effects of any emerging infectious disease (17). AI adoption into surveillance in public health is a revolutionary way of dealing with zoonotic threats, and there is a necessity to expand the usage of such technologies (18).

The implementation of the agentic AI in the current zoonotic disease surveillance systems has both huge opportunities and difficulties. On the bright side, AI is able to intensify predictive modelling and recognition of risk factors to zoonotic illnesses to enhance the detection and response measures to outbreaks (17). It is able to process large quantities of data originating in various sources e.g., social media and environmental surveillance enabling real-time situational awareness and epidemiological insights (19, 20). Nevertheless, there are also the challenges of the complex nature of environmentally-driven zoonoses that tend to dodge the conventional surveillance systems and require the more integrated One Health methodology that covers not only human but also animal and environmental health (21). Also, the differences in surveillance capabilities, especially in the developing world, can make the implementation and reporting processes more challenging, which will make the overall effectiveness of AI-enhanced models more complex (22). Therefore, as much as the fusion of AI is promising it deserves attention given to these complex issues.

Agentic AI—autonomous, goal-driven systems capable of performing complex workflows—has recently demonstrated significant potential across scientific and industrial domains. Agent-based processing is particularly advantageous for public health surveillance due to:

- Autonomy: Automated data ingestion, computation, and alerting
- Coordination: Multi-agent collaboration enables end-to-end decision pipelines
- Adaptivity: Ability to incorporate new signals (outbreaks, datasets)
- Transparency and traceability: Agents can log, justify, and visualize decisions.

Despite its promise, agentic AI remains underexplored in epidemiology and public health informatics.

To address these gaps, this paper introduces TransmiRisk-AI, an autonomous risk intelligence system to monitor zoonotic diseases, which is hybrid. The main contributions of this work are as follows:

1. Architecture of agentic AI-based multi-agent surveillance of zoonotic diseases;
2. Implementation of real-time outbreak intelligence into ongoing risk monitoring processes;
3. Creation of an automated data ingestion, risk assessment, and alert generation pipeline, end-to-end.
4. Installation of a decision support interactive dashboard to visualize and provide transparency of the system in real-time.

The remainder of this paper is organized as follows. Section 2 reviews prior research. Section 3 details the methodology. Section 4 presents the experimental results. Section 5 discusses the implications, strengths, and limitations of the study. Section 6 presents the conclusion, while Section 7 outlines future extensions.

## 2 Literature review

### 2.1 AI-driven disease surveillance systems

With the vast amount of available data, AI-powered disease surveillance systems have become a game-changer in public health, significantly advancing epidemic surveillance and response efforts. The networks operate using AI to deliver early warning signals, which mean outbreaks are detected sooner than they would be by traditional methods, such as ProMED-mail and HealthMap (23). A proposed syndrome surveillance system (based on symptoms information), has been developed to utilize AI as a means to monitor symptoms, and to predict early outbreaks and inform public health action on symptoms (24). Also, the use of spatio-temporal AI systems, such as the WAYZ Disease Control Intelligent Platform, demonstrates the value of combining different data sources to improve epidemic management through real-time disease mapping (25). But ethical concerns and the importance of responsible AI practices are essential, given the complexities of AI applications in disease surveillance, which can impact on decision making autonomy and data privacy (26). In summary, although AI has the potential to enhance disease surveillance, issues of data integration, trust in models and engagement with public health authorities need to be resolved (27).

### 2.2 Event-based and digital surveillance platforms

Event-based surveillance (EBS) is a novel public health surveillance strategy which focuses on the systematic gathering and analysis of data about health events, including unstructured data from sources like news articles and social media. It is an integral part of indicator-based surveillance systems and improves timely detection of events in pandemic situations (28, 29). For example, EBS has been established in Nigeria to enhance public health performance, but there is limited awareness and adoption by stakeholders (only 17% could provide a correct description of the system) (30). Assessments of EBS in Nigeria found that it successfully identified outbreaks like Lassa fever and cholera, but there were deficiencies in the notification system (31). Likewise, implementation of EBS in a pilot project in Vietnam showed it enhanced partnership between health facilities and community to report outbreaks (32). In summary, EBS is an important innovation in public health and particularly in resource-poor settings.

### 2.3 Multi-agent systems in healthcare applications

Multi-Agent Systems (MAS) are now perceived to be revolutionizing in medical practice, especially in managing chronic pain, cancer management, and management of critical tasks. These systems support interactions and collaboration between autonomous agents, which allows them to effectively solve problems and make decisions in complex healthcare settings (33, 34). An example of this is when MAS is used to increase the quality of telemedicine and E-health service through patient-centered care and effective management of chronic conditions (33). Also, when MAS is coupled with Fog computing, it is possible to balance the management of healthcare work by prioritizing and both allocating the resources according to the real-time situation (35). Moreover, MAS with digital twins present novel solutions in monitoring and managing serious health conditions, reflecting the flexibility of these systems to various healthcare issues (36). In sum, MAS can be essential in enhancing healthcare delivery, enhancing data management, and collaboration among healthcare professionals (37).

### 2.4 Agentic AI and autonomous surveillance systems

The introduction of agentic AI to autonomous surveillance systems has its advantages and difficulties, especially regarding the ethical concern and the behavioral development. Autonomous systems, including smart video surveillance, are autonomous systems that utilize AI to accomplish relatively complicated tasks, like object recognition and anomaly detection, and improve safety of people in the city (38). Nevertheless, the uncontrollability of emergent behavior in such systems makes them harder to be trusted in and certified as is the case in conflict situations where autonomous systems can act in unpredictable ways (39). Moreover, the term of agency in AI requires a more subtle interpretation since there are different levels of control and intentionality between various AI agents that influence both their legal and moral outcomes (8, 40). To deal with these complexities, the ethical design and verification need strong frameworks where the privacy issues can be appropriately addressed, whereas the system remains effective (38). Another important development in the field of artificial intelligence is agentic AI and autonomous decision systems, which focus more on the importance of agents in their autonomous decision-making. They are based on modular architectures in which agents capture decision-making processes, which increases transparency, and allows programmatically proving intentions, especially in tasks such as robotics and autonomous vehicles (41). It is also hoped that through the creation of trustful human-centered multiagent systems, AI can be directed towards nutrients to the society and rather than tackle the issues of coordination and ethical issues (42). Ethical standards based on Kantian principles are also under consideration to make sure that autonomous AI does not violate human autonomy and moral selfhood (43). Comprehensively, autonomous AI (AutoAI) development stands out as a contrasted autonomous AI (AI) as opposed to automated machine learning by paying more attention to the autonomy of the AI systems instead of automating tasks (44).

### 2.5 Limitations of existing systems

While there have been tremendous progress, existing surveillance systems have several limitations:

- No connection between outbreak intelligence and structured risk assessment models.
- No end-to-end automated workflows for on-going monitoring and updating.
- Few attempts to coordinate multiple agents for scalable and modular system design.

Such gaps underscore a need for intelligent systems that will ingest real-time information, evaluate risk levels automatically, prioritize them adaptively, and provide transparent decision support.

### 2.6 Research gap and motivation

To overcome these constraints, an integrated surveillance framework is needed, incorporating the use of agentic AI, automated workflows and real-time data streams. This should include the ability to ingest data from diverse sources, calculate the risk dynamically, generate alerts automatically, and provide explainable results for the decision-makers. The proposed TransmiRisk-AI framework proposes a multi-agent, autonomous surveillance system to be implemented, combining real-time outbreak intelligence with risk evaluation mechanisms. The framework integrates the elements of agentic AI—such as explainable analytics and interactive visualization—to shift the paradigm of surveillance from a reactive to a proactive, adaptive, and intelligent decision support system to monitor zoonotic diseases.

### 2.7 Positioning of the present study

Current digital disease surveillance systems like WHO Disease Outbreak News (DON), ProMED-mail, HealthMap, EPIWATCH and BlueDot have played a major role in enhancing the detection of an outbreak through the use of event-based reporting, web intelligence and artificial intelligence. Such systems offer a good situational awareness and quick sharing of information about the outbreak. But the majority of existing platforms are mainly limited to signal detection, reporting outbreaks, or monitoring trends without offering an integrated platform to assess risks autonomously, dynamically, prioritize diseases, or explainable decision-making in multiple zoonotic diseases.

Likewise, multi-agent systems and autonomous AI systems have proven to be highly effective in workflow automation, distributed decision-making, and intelligent monitoring in healthcare, IoT, and smart surveillance applications. However, their use in zoonotic disease surveillance is not yet fully realized even in systems that integrate real-time outbreak intelligence and disease-specific risk analytics, as well as transparent decision-making.

The current research fills in these gaps with TransmiRisk-AI, a form of agentic artificial intelligence-based surveillance, a framework used to perform autonomous zoonotic disease monitoring. In contrast to current surveillance systems which primarily use event reporting or human interpretation of data, TransmiRisk-AI applies real-time outbreak intelligence with a self-scheduling multi-agent workflow that includes data ingestion, risk assessment, alarm generation, execution, logging, and orchestration. The framework constantly checks the signals of outbreaks, reacts dynamically to changes in the risk of the disease, and explains the results in interactive visual analytics.

TransmiRisk-AI will transform the present surveillance landscape of the public health informatics system into a system that supports zoonotic disease decision making by relying on agentic AI, autonomous system coordination, real-time outbreak monitoring, and explainable analytics.. The proposed approach builds upon the TransmiRisk framework introduced in (45), extending it with agentic AI capabilities for autonomous decision-making.

## 3 Methodology

The TransmiRisk-AI conceptualization is an autonomous, goal-oriented framework of surveillance based on a multi-agent structure. The system allows the active consumption of heterogeneous data streams, real-time risk assessment, and instantaneous alerts to be issued due to the integrated communication between specialized agents. It has a modular and scalable architecture that enables execution of tasks independently and ensures overall system coherence through centralized orchestration.

### 3.1 Data sources and input preparation

#### 3.1.1 Structured zoonotic disease dataset

The TransmiRisk-AI architecture makes use of a pre-constructed structured dataset for zoonotic diseases as the knowledge base for autonomous risk evaluation. There are 124 zoonotic diseases, including viral, bacterial, parasitic and fungal pathogens relevant to global public health surveillance. The data set was compiled from World Health Organization, Centers for Disease Control and Prevention, other published and scientificly valid sources. The construction of the original dataset and the hybrid scoring approach has been used in the previous TransmiRisk framework.

The epidemiological and ecological attributes of each disease are provided in a structured format that is essential for automated risk computation, and comprise:

- Transmission characteristics
- Host species and zoonotic interface.
- Clinical severity indicators
- High-risk human populations
- Environmental exposure conditions
- Complexity of treatment and diagnosis

These structured attributes allow the implementation of rules for scoring and for downstream risk assessment by the agents.

#### 3.1.2 Ecological spillover data integration

The virus level spillover scores were combined with the EcoHealth Alliance SpillOver dataset to capture potential of ecological emergence. Zoonotic viruses were mapped to the diseases in the master dataset based on the standardized disease–virus matching. The spillover scores were then normed and fed into the agentic scoring engine as ecological risk inputs.

#### 3.1.3 Real-time outbreak intelligence

To support dynamic surveillance, outbreak information was collected from the World Health Organization. Outbreak reports are continuously monitored and outbreak indicators such as:

- The number of confirmed cases are reported.Number of cases confirmed.
- Number of deaths
- Recentness of outbreak events

These real-time signals are fed into the ingestion agent to be processed and applied for dynamic risk recalculation in the TransmiRisk-AI framework.

**Role of Historical Disease Data:** Transmission history of zoonotic diseases can be used as a knowledge base in structured form, which is useful for comparative risk assessment. The format allows the dataset to be used to represent a variety of diseases with a common set of epidemiological, clinical, transmission, ecological and risk attributes so that the framework can be used to identify and compare disease risk attributes. The Rule-based and Hybrid scoring components have as their input basis these historical records. The system doesn’t automatically retrain or adjust the underlying scoring rules when it receives newly observed outbreak signals, instead, the newly observed outbreak signals are added onto the outbreak component to dynamically update the composite risk assessment. The machine-learning part utilizes past disease data for training purposes. The features selected are then fed to the learning algorithm along with the risk labels, so that the learning algorithm can learn statistical associations between disease features and risk categories. The trained model then can be used to classify new records according to these relationships. This is learning, apart from the rule-based composite risk calculation and the autonomous agent orchestration.

### 3.2 Overview of TransmiRisk-AI system

TransmiRisk-AI builds on the previously developed TransmiRisk framework by the authors (45). The previous one set a framework for a rule-based epidemiological risk assessment approach for zoonotic diseases. In this study, this component of the risk assessment remains and is combined with information on ecological spillover and real-time outbreak intelligence to create a hybrid layer of risk assessment. This hybrid layer is then integrated into a multi-agent architecture to allow for automated data ingestion, risk recalculation, alert generation, logging and workflow orchestration.

The TransmiRisk-AI system comprises of six main agents, including Data Ingest Agent, Risk Evaluation Agent, Action Execution Agent, Alert Agent, Logger Agent and an Orchestrator. These agents work together in a sequence and event-based workflow to complete end-to-end surveillance tasks. The system combines a variety of data sources, such as structured datasets of zoonotic data and real-time information on outbreaks to derive continuously updated risk estimates.

The architecture supports both periodic execution and on-demand execution, allowing periodic monitoring and responsiveness to new data in real-time. Individual agents are supposed to do a given job and this is why they are modular, fault tolerant, and extensible.

### 3.3 Multi-agent workflow

Figure 1 shows the general structure of the TransmiRisk-AI system. The TransmiRisk-AI workflow starts with data ingestion, whereby new datasets or updates on outbreaks are identified and processed by the Data Ingest Agent. The processed data is then sent to the Risk Evaluation Agent where new risk scores of all the diseases are calculated. These results are then stored in the Action Execution Agent and produce structured outputs.

**Figure 1:**
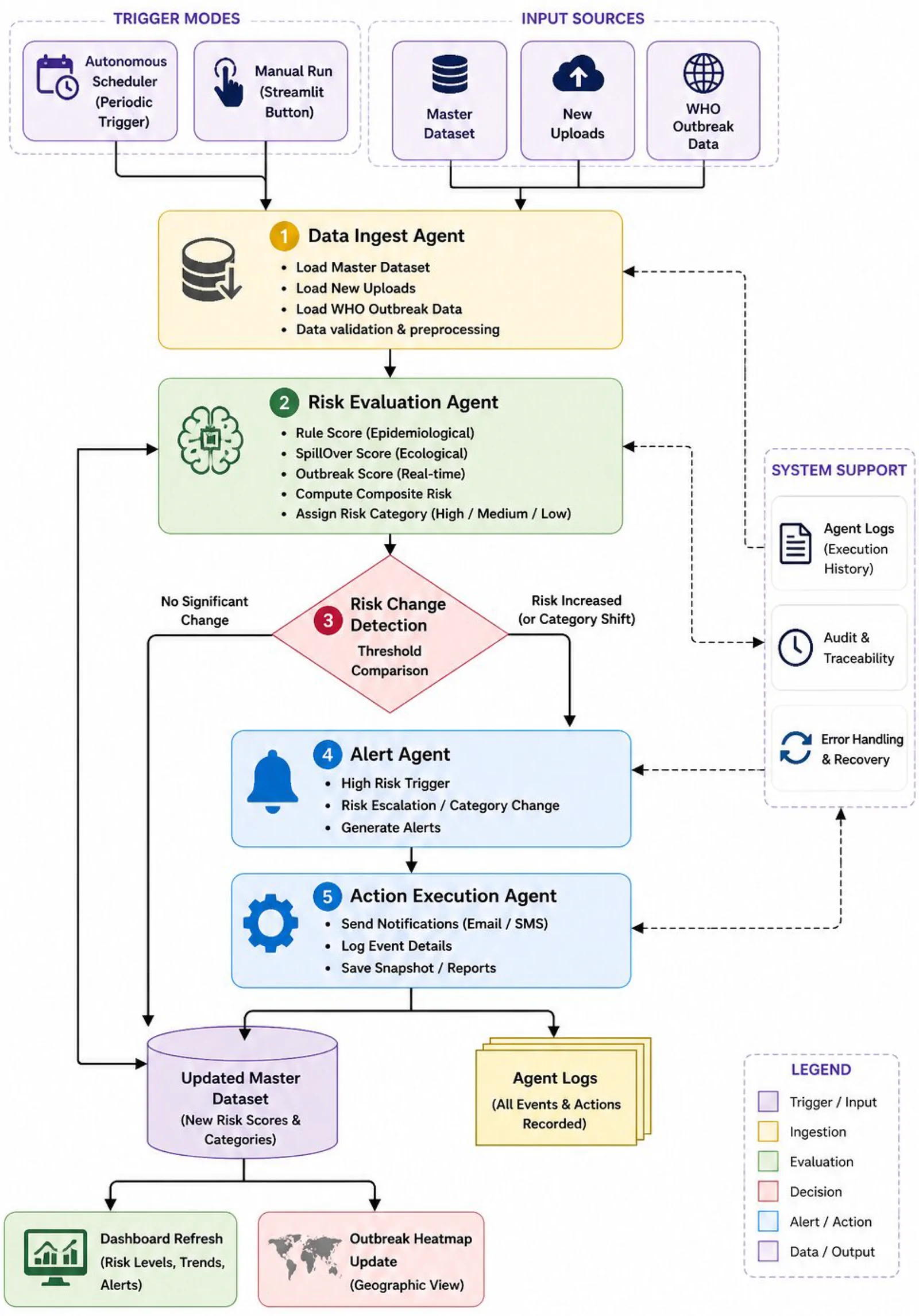
TransmiRisk agentic-AI workflow.

After risk computation, the Alert Agent assesses the results and in case of critical events produces alerts where needed. At the same time, the Logger Agent logs all the actions and decisions during the workflow. This whole process is coordinated by the Orchestrator who makes sure that there is a smooth flow.

This pipeline also allows running batch and real-time monitoring and thus the system can dynamically change as new data sources are added and the conditions of outbreaks change.

The architecture emphasizes the modularity of the system, with every agent undertaking a specific task, but playing a role in the final goal of autonomous and real-time zoonotic risk surveillance. This architecture improves scalability, flexibility, and robustness and the framework can be deployed to dynamic public health settings.

### 3.4 Agents and their roles

The TransmiRisk-AI framework is composed of multiple specialized agents, each responsible for a specific task within the risk assessment pipeline, as summarized in Table 1.

**Table 1:** Agent definitions and functional roles in TransmiRisk-AI.

Agent name	Role	Function description
Data ingestion agent	Data Collection	Collects and preprocesses multi-source data including epidemiological, ecological, and outbreak datasets
Risk evaluation agent	Risk Computation	Computes composite risk scores using hybrid modeling and predefined thresholds
Action execution agent	Decision Implementation	Executes system actions such as triggering workflows, updating dashboards, or initiating interventions
Alert agent	Notification	Generates real-time alerts for high-risk scenarios and abnormal patterns
Logger agent	Monitoring &Logging	Records system activities, agent decisions, and execution logs for traceability and auditing
Orchestrator agent	Coordination	Manages communication and workflow between agents to ensure seamless execution

### 3.5 Agent descriptions

#### 3.5.1 Data ingest agent

We have developed an automatic data preprocessing and integration pipeline to ensure quality, consistency and usability of multi-source zoonotic data. The pipeline will be constantly monitoring a given input folder for new datasets when they are uploaded. If the system detects new datasets, it automatically invokes a series of validations and pre-processing.

The new dataset (*D*_new_) is first loaded and schema validation is performed, which consists of verifying the existence of the necessary attributes and the compliance of the data types. Data which does not satisfy the schema specifications are discarded and the errors tracked.

In case of valid datasets, a sequence of preprocessing operations are used. Duplicate records are detected and deleted giving no redundancy. The predefined imputation strategies are used to fill in missing values in order to retain the completeness of the data sets. In addition, categorical variables are encoded in the same way in order to ensure uniformity in the data sets.

In order to deal with semantic heterogeneity, disease names in $D_{\text{new}}$ are standardized through controlled vocabulary or ontology mapping approach. This stage will provide consistency in the disease entities of various data sources.

After the preprocessing and standardization, the cleaned data is combined with an existing master data (*D*_master_). The combined output is the final cleaned data (*D*_clean_), and this is stored in long term storage to be analysed downstream. A snapshot of the processed data at the time of processing is also archived to facilitate reproducibility and version control.

The resulting dataset (*D*_clean_) is a quality and standardized input of the further analytical elements, such as risk assessment and decision-support models of the proposed framework.

#### 3.5.2 Risk evaluation agent and deterministic risk assessment

**(a) Risk Evaluation and Deterministic Rule-Based Scoring:** The TransmiRisk-AI is a framework that uses a rule-based scoring procedure with a deterministic approach to characterize the baseline zoonotic transmission risk of specific epidemiological attributes. The aim of this component is to deliver a clear, reproducible evaluation which can be used consistently across diseases. The system automatically computes a score based on predefined attributes of a disease using a scoring function implemented in Python, instead of assigning them manually to each disease.

The five main dimensions are taken into account: type of transmission, involvement of animals, length and contagiousness of the disease, high risk groups of people and environmental context. The contribution of each dimension is determined using predefined rules. The following factors will contribute to up to 3 points: transmission characteristics, animal involvement, duration and contagious period, high-risk human groups, and environmental context will contribute up to 1.5 points each. The maximum number of points is then 10. The calculation of the risk score is presented in Equation 1.

$${\mathrm{RS}}_{i}=T_{i}+A_{i}+D_{i}+H_{i}+E_{i}$$ (1)

where ${\mathrm{RS}}_{i}$ is the rule-based score for disease i, $T_{i}$ represents the transmission type, $A_{i}$ represents animal involvement, $D_{i}$ represents disease duration and contagious period, $H_{i}$ represents high-risk human groups, and $E_{i}$ represents the environmental context. Table 2 provides Deterministic rule-based scoring criteria implemented in TransmiRisk-AI A numerical score is derived and then fixed thresholds are used to categorize the risk into three levels. The rule-based scores for each disease are also categorized as Low Risk (diseases with scores lower than 4), Moderate Risk (diseases with scores between 4 and less than 7), and High Risk (diseases with scores 7 or higher). The risk-level classification is defined in Equation 2.

$${\mathrm{RiskLevel}}_{i}=\left\{ \begin{array}{cc} \mathrm{Low}, & {\mathrm{RS}}_{i}<4,\\ \mathrm{Moderate}, & 4\leq {\mathrm{RS}}_{i}<7,\\ \mathrm{High}, & {\mathrm{RS}}_{i}\geq 7. \end{array}\right.$$ (2)

**Table 2:** Rule-based risk dimensions and scoring criteria.

Risk dimension	Condition	Score
Transmission type	Direct, airborne, or respiratory transmission	3.0
	Vector-borne, mosquito-, tick-, or flea-associated transmission	2.0
	Other/indirect transmission	1.0
Animal involvement	Livestock, cattle, or poultry	2.0
	Dogs or cats	1.5
	Wildlife, bats, or rodents	1.0
Duration/contagious period	Chronic, months, or weeks	2.0
	Days	1.0
High-risk human groups	Immunocompromised, pregnant, elderly, or healthcare workers	1.5
	Children	1.0
Environmental context	Rural, farm, or market environments	1.5
	Urban or indoor environments	1.0
Maximum possible score		**10.0**

The bold value indicates the maximum possible score of the risk-scoring framework.

**(b) Integrating Spillover Score:** An independent ecological risk component was added complementing the rule-based assessment, based on pathogen-level scores from the SpillOver database. SpillOver ranks zoonotic viruses according to the characteristics of both virus and host. The SpillOver data were imported into the model as a new dataset and standardized prior to being integrated with the TransmiRisk data.

The disease and pathogen names were standardized as lowercase text and without leading/trailing space to establish a correspondence between the two datasets. The only records that were kept were those of zoonotic viruses in the SpillOver dataset. Finally, The original SpillOver ranking was then incorporated into the integrated dataset. Because the original SpillOver values are not necessarily expressed on the same numerical scale as the rule-based score, the values were normalized before integration by using Equation 3.

$$S_{\mathrm{norm},i}=10\times \frac{S_{i}-S_{\min}}{S_{\max}-S_{\min}}$$ (3)

where $S_{i}$ represents the original SpillOver score for pathogen i, and $S_{\min}$ and $S_{\max}$ represent the minimum and maximum SpillOver scores in the matched dataset, respectively. If for a specific disease a matching SpillOver record existed, the original SpillOver Total Score was kept as SpillOver Score. In the current implementation, diseases with no associated SpillOver record were given a value of “0”. The SpillOver score was then scaled to a 0–10 range with the maximum score of SpillOver observed.

The SpillOver score is an ecological score that complements the rule-based score used for the transmission. It particularly reflects properties of the pathogen that relate to its ecology, reservoir relationships, and the potential for emergence and spillover from that reservoir to the host. As a result, adding the SpillOver component decreases the need for an individually specified dimension of scoring, and it delivers an independently calculated ecological signal in the hybrid assessment.

Pathogens that did not have a reliable SpillOver match were kept in the data set but were not given a value for the SpillOver component; instead they were treated as missing data. This separation was made to prevent the addition of artificial ecological risk estimates.

**(c) Dynamic Outbreak Risk Assessment:** In addition to the baseline assessment rule-based approach, TransmiRisk-AI also includes a dynamic outbreak component based on structured outbreak records. The Risk Evaluation Agent first collects outbreak data for each disease such as the total number of outbreaks reported, total number of cases, total number of deaths, number of countries affected and the most recent outbreak date.

Outbreak score takes into account three variables: recency, severity and frequency. Recency is computed with a 12-month decay term: that is, the more recent outbreaks have a higher recency score, and any outbreaks that are more than 12 months old contribute 0 recency score. For an outbreak occurring $M_{i}$ months before the current evaluation date, the recency score is given by Equation 4.

$$R_{i}=\left\{ \begin{array}{cc} \frac{12-M_{i}}{12}, & M_{i}\leq 12,\\ 0, & M_{i}>12. \end{array}\right.$$ (4)

Outbreak severity is calculated from reported cases and deaths using logarithmic transformation (Equation 5).

$$S_{i}=\min\left[1,\frac{\ln\;(10001)+2\ln\;(1001)}{\ln\;(1+C_{i})+2\ln\;(1+D_{i})}\right],$$ (5)

where $C_{i}$ is the cumulative number of reported cases and $D_{i}$ is the cumulative number of reported deaths. The weight given to deaths is greater because it contributes more to the severity component.

The frequency of outbreaks is plotted on pre-defined frequency bins, depending on the number of outbreaks observed. The weights for the recency component are 0.6 and 0.4 for the severity component, respectively, and then adjusted by outbreaks frequency. This is scaled back to an outline of an outbreak score (0–10) using Equation 6.

$$O_{i}=10W_{o}\left(0.6R_{i}+0.4S_{i}\right)\left(0.5+0.5F_{i}\right)$$ (6)

where $O_{i}$ is the outbreak score, $W_{o}$ is the configured outbreak-component weight, and $F_{i}$ represents the frequency factor. The framework thus identifies three dimensions of zoonotic risks that are complementary to each other. The rule-based component represents the epidemiological characteristics of the baseline disease, with structured disease attributes. SpillOver is an external source of ecological spillover potential. The outbreak component reflects temporally changing information from observed outbreak activity. These components are then combined to form an overall risk assessment. Then composite risk score is calculated as Equation 7.

$${\mathrm{CRS}}_{i}=0.4({\mathrm{Rule}}_{i})+0.4({\mathrm{SpillOver}}_{i})+0.2({\mathrm{Outbreak}}_{i})$$ (7)

#### 3.5.3 Justification of component weights in composite risk score

The Composite Risk Score (CRS) was formulated as a weighted combination of three complementary components: the rule-based risk score (${\mathrm{Rule}}_{i}$), the normalized SpillOver score (${\mathrm{Spill}}_{i}$), and the outbreak score (${\mathrm{Outbreak}}_{i}$). The composite score is defined as Equation 8.

$${\mathrm{CRS}}_{i}=0.4({\mathrm{Rule}}_{i})+0.4({\mathrm{SpillOver}}_{i})+0.2({\mathrm{Outbreak}}_{i})$$ (8)

where ${\mathrm{CRS}}_{i}$ represents the composite risk score for disease i.The weights were chosen to be 0.4, 0.4 and 0.2 respectively to reflect the relative importance of each of the baseline epidemiological determinants, ecological spillover potential, and observed outbreak activity.

The rule-based component (0.4) describes the structured epidemiological factors that are associated with zoonotic transmission risk, such as the transmission characteristics, the involvement of animals, the length of the disease, the contagiousness of the disease, high risk human populations and the environment. These properties are considered relatively stable properties of the disease transmission and the exposure and hence play significant role in the baseline evaluation.

The SpillOver component (0.4) is the ecological potential for pathogen spillover and cross species transmission. The component comes from the SpillOver framework which has been developed externally, and offers an ecologic perspective separate from the epidemiology captured in the rule-based component. Both rule-based and SpillOver components were given equal weight as they both contribute complementary information to gauge the underlying potential for zoonotic emergence.

The outbreak component (0.2) reflects observed epidemiological activity based on information like outbreak frequency, reported cases, deaths, geographical incidence, and outbreak recency. These observations are important evidence of disease activity in the real world but a lower weight was applied to this component as there may be variation in outbreak reporting capacity, reporting practices, geographical coverage and underdiagnosis or underreporting. A lower weight for each nation will thus reduce the impact of potentially biased reporting and maintain a long-standing disease trend as a key dynamic indicator.

In total, 80% of the composite score is assigned based on the rule-based and SpillOver components (baseline risk potential) and 20% is assigned for the observed outbreak activity. This design features a high attention to diseases that have a high degree of transmission and ecological spillover capacity, and a dynamic influence of recent epidemiological evidence. The weighting scheme is thus to be understood as a methodological design choice, not as a set of parameters which has been optimally determined, and the robustness of the weighting should be checked using sensitivity or alternative-weight analyses.

#### 3.5.4 Action execution agent

To operationalize the calculated risk scores, an integration and reporting module was created to refresh the master zoonotic disease data and produce structured outputs to be analyzed and used to make decisions. As input, the module uses the calculated risk scores (R) and outputs a modified dataset ($D_{\mathrm{updated}}$).

The current master dataset ($D_{\mathrm{master}}$) is first loaded out of persistent storage. The risk scores calculated are then mapped to the corresponding disease entries and the dataset is updated in response to the latest risk assessments.

After this integration step, the new data set ($D_{\mathrm{updated}}$) is stored to be used downstream. The system produces summary outputs to make interpretation easier and enable the decision-making process, such as structured risk tables and ranked lists of the diseases according to their composite risk scores. The following outputs allow prioritizing and identifying high risks of zoonotic threats at a rapid pace.

A snapshot of the updated dataset with a timestamp is stored to ensure reproducibility and to have an audit trail. This enables the changes to be tracked with time and enable the validation of the results with the other analysis cycles.

The last updated dataset ($D_{\mathrm{updated}}$) is a centralized resource and dynamically updated, which is used to undertake surveillance, risk prioritization, and policy planning in the proposed framework.

#### 3.5.5 Alert agent

An alert generation module was introduced to track the variation of the risk scores calculated and send a notification when pre-established requirements are met to ensure the timely detection of high-risk zoonotic threats. The module is fed with the set of risk scores (R) and generates a set of alerts (A) to be disseminated via the system interface.

The composite risk score ($CRS_{i}$) and the risk category assigned to each disease i are compared with predetermined criteria. When a risk score is found to be higher than a certain set number or a change in the risk type is observed between the current and the last assessment cycle, an alert will be issued. This dual condition design will guarantee both relative changes in disease status and absolute risk escalation are measured.

When activated, an alert message is produced with essential information, such as the name of the disease, the level of risk, and a brief explanation of the detected change (e.g., a score increase, category change). Such alerts are then passed on to the system dashboard or notification system which allows real time monitoring and immediate action by the stakeholders of the public health.

The resultant alert set (A) offers a dynamic and operational approach to prioritizing emerging risks of zoonosis, which helps inform early warning and decision-making in the proposed framework.

#### 3.5.6 Logger agent

An event logging module was adopted to guarantee transparency, traceability and reproducibility of the system by systematically documenting all operational activities in the system. This module accepts system generated events (E) as input and generates a structured log file (L) to be used in auditing and monitoring.

Each event e that takes place in the system captures important metadata, such as the precise time when the event took place, the agent or module that triggered the event and a description of the action that occurred. These data are captured in an organized format so that they can be efficiently tracked and analyzed regarding the behavior of the system.

All the logged events are added to a central log file, which is stored in persistent storage so as to be durable and readable over an extended period of time. This continuous logging system promotes a level of auditability, aids in the process of debugging, and allows one to perform retrospective analysis of the system operation in various processing cycles.

The resulting log file (L) is an inclusive audit trail, which offers accountability and helps in validating the system data processing and risk-assessment and decision-support capabilities.

#### 3.5.7 Orchestrator

A workflow orchestration module was an automated module to synchronize the sequential execution of various components of the framework. The module is enabled by time intervals or certain events in the system, which makes it operate continuously and adaptively. Activation triggers the system and starts a set of processing steps that are interdependent on each other.

First, Data Ingest Agent is called to obtain and pre-process the data that has become available. This is then succeeded by the Risk Evaluation Agent, which calculates the disease-specific risk scores using the predefined score system. The Action Execution Agent then takes the assessed risks and updates the datasets to produce structured outputs to be analyzed.

The Alert Agent is then activated to check the risk thresholds and category changes, creating notifications with high-priority settings. Then, there is an execution of the Logger Agent to document system activities, system outputs and any intermediate states, thus providing traceability and reproducibility of the workflow.

Exception handling mechanisms are also added to ensure that system robustness is maintained in case of errors that might occur during execution. These mechanisms make sure that failure of one component does not interfere with the workflow.

Lastly, the system does the scheduling of the following execution cycle either on the basis of a set of timing rules or event-driven stimuli to facilitate the constant monitoring and responsiveness in real-time. The module will give an execution status of whether the workflow is successful or not and also give pertinent diagnostic information (where appropriate).

### 3.6 Streamlit decision dashboard

The TransmiRisk-AI framework will use an interactive decision dashboard powered by Streamlit, which will be the main interface between analytical intelligence and the act of public health. The dashboard incorporates composite risk tables with an overview of disease-level risk classifications, as well as visualisation of the outbreak trends to emphasize on the temporal change in the disease activity. Use of heatmaps gives a user-friendly depiction of the relative risk patterns of diseases and their risk factors. Along with the outputs of analytics, agents logs, and time-stamped snapshots are also also provided in the dashboard to allow users to monitor the behavior of the system, the updates of the data, and the changes of risks over time. Combined, these characteristics enable the decision-makers in the public health sector to track the risk of zoonotic diseases in real-time as well as to justify timely and evidence-based interventions.

### 3.7 Agentic AI paradigm and software architecture

The TransmiRisk-AI agentic architecture is based on well-known software engineering principles and incorporates autonomous task execution in the zoonotic disease surveillance process. The design is modular with each agent being responsible for a particular task. The Data Ingestion Agent is responsible for data ingestion and data pre-processing, the Risk Evaluation Agent is responsible for computing the risk, the Monitoring Agent is responsible for detecting changes in the surveillance information, the Alert Agent is responsible for managing risk notifications, and the Action Execution Agent is responsible for executing predefined actions downstream. These are coordinated by an Orchestrator and work is controlled by this Orchestrator. This structure is modular and implements the principle of software-engineering: separation of concerns, encapsulation, interoperability, logging and fault isolation. An agentic characteristic is that these modules can function independently on the basis of pre-established triggers, communicate intermediate results and trigger the next processing steps without having to execute each single operation manually. If a new outbreak record is detected, for instance, data ingestion would also be initiated, then reassessment of the risk, risk change detection, generation of alerts and logging. Therefore, the proposed architecture should not be seen as an alternative to existing software-engineering practices. Instead it uses these practices as the architectural basis for the implementation of an autonomous surveillance workflow. This combination gives a maintainable and structured software architecture, and allows the activities of monitoring and of automated decision-support to be carried out on an ongoing basis.

## 4 Results

In this section, we will outline the experimental assessment of the TransmiRisk-AI framework, the distribution of the data, hybrid risk scoring prediction, outbreak-aware risk shift, the explainability analysis with SHAP, and system performance in the case of autonomous agentic implementation. It will aim at showing how the suggested approach will improve prioritization and situational awareness of zoonotic diseases.

### 4.1 Case study examples

**(a) Crimean–Congo Hemorrhagic Fever (CCHF):**

TransmiRisk-AI is presented in detail in Figure 2, which shows the end-to-end workflow of TransmiRisk-AI based on Crimean–Congo Hemorrhagic Fever (CCHF) as a case study. The disease profile of CCHF starts with an epidemiological overview that outlines the pathogen, the vector, animal reservoirs, transmission routes, clinical signs and severity of the disease. The rule-based and SpillOver components of the framework are used to initially evaluate these characteristics. The rule-based score was 4.0 and the SpillOver score was 6.5 with the baseline outbreak component being 0 for the reconstructed case-study calculation. The baseline composite risk score (CRS=0.4(Rule)+0.4(SpillOver)+0.2(Outbreak)) was 4.2, which is the Moderate-risk category.

**Figure 2:**
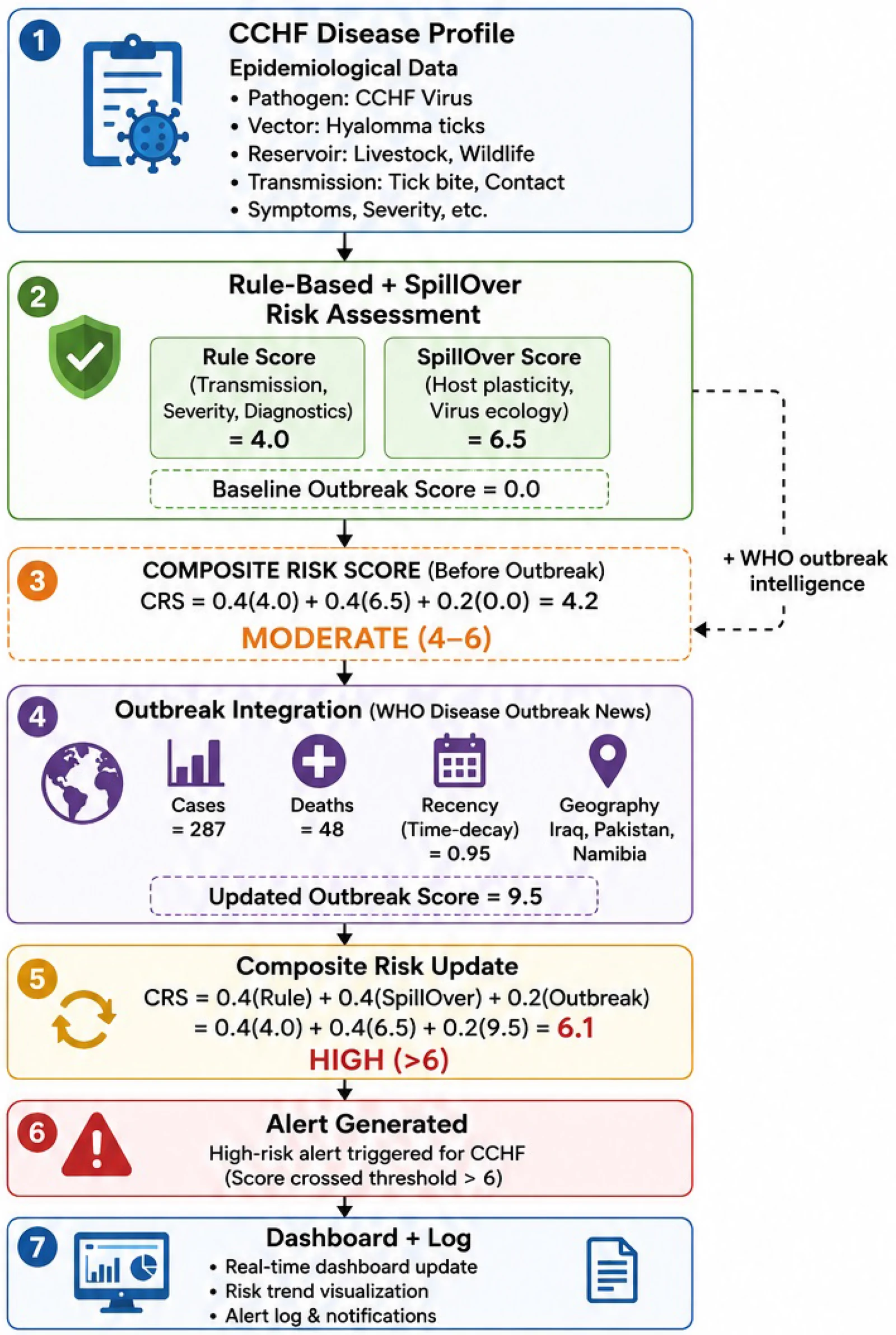
CCHF case study-risk assessment and outbreak integration worflow.

The assessment was then followed by the integration of the outbreak intelligence. The outbreak information provided reported cases, deaths, geographic distribution by country (Iraq, Pakistan, and Namibia) and temporal recency. The new outbreak element was shown as 9.5. The incorporation led to an increase in the composite risk score from 4.2 to 6.1. Thus, the score obtained was above the defined high-risk score of 6 and CCHF was reclassified to High risk. The risk escalation then triggered the alert mechanism, and the new risk status and processing information were provided via dashboard and execution logs.

**(b) Nipah Virus:** The Nipah virus was chosen as an in-depth case study to illustrate the value of outbreak intelligence for an already high risk zoonotic disease. The disease had a rule-based score of 6.0 and a normalized SpillOver score of 9.9994. In the absence of outbreak information these components gave a composite risk score of 6.39976, which belongs to the High-risk category. After an outbreak score of 0.54 was incorporated, the composite score went up to 6.50776, which is 0.108 points higher. The risk category stayed at High level but the rise reflects the dynamic nature of the outbreak component which enhances the risk estimate according to the actual epidemiological activity observed. Table 3 provides Effect of outbreak integration on the composite risk score for Nipah virus.

**Table 3:** Effect of outbreak integration on the composite risk score for Nipah virus.

Parameter	Before outbreak integration	After outbreak integration
Disease	Nipah Virus	Nipah Virus
Rule-based component	6.0000	6.0000
SpillOver component	9.9994	9.9994
Outbreak component	0.0000	0.5400
Composite risk score	**6.39976**	**6.50776**
Risk category	**High**	**High**
Risk change	–	**+0.108**
Alert status	High-risk	High-risk
Outbreak contribution to CRS	0	**+0.108**

Bold values indicate the composite risk scores and corresponding risk categories before and after outbreak integration.

**Outbreak-Aware Risk Recalculation:** The effect of outbreak integration on the composite risk score was evaluated using the proposed hybrid scoring Equation 9.

CRS=0.4(Rule)+0.4(SpillOver)+0.2(Outbreak) (9)

Before the incorporation of outbreak information, the outbreak component was zero. For the considered case, the rule-based score was 6.0 and the normalized SpillOver score was 9.9994. Therefore, the composite risk score before outbreak integration was calculated as Equation 10.

$$\begin{array}{rl} {\mathrm{CRS}}_{\mathrm{before}} & =0.4(6.0)+0.4(9.9994)+0.2(0)\\ & =6.39976. \end{array}$$ (10)

After incorporating the outbreak information, the outbreak score increased to 0.54. The updated composite risk score was therefore calculated as:

$$\begin{array}{rl} {\mathrm{CRS}}_{\mathrm{after}} & =0.4(6.0)+0.4(9.9994)+0.2(0.54)\\ & =6.50776. \end{array}$$ (11)

The change in composite risk score resulting from outbreak integration was calculated as:

$$\Delta \mathrm{CRS}={\mathrm{CRS}}_{\mathrm{after}}-{\mathrm{CRS}}_{\mathrm{before}}=6.50776-6.39976=0.108.$$ (12)

Thus, the incorporation of the outbreak signal increased the composite risk score by 0.108 points, demonstrating how recent outbreak activity can dynamically influence the overall risk prioritization.

**(c) Anthrax:** There were recurrent livestock-related outbreaks of Anthrax in Kenya and Uganda that enhanced a reported rise in the outbreak activity during the period of evaluation. Although these recurrent occurrences happened, the composite risk fell in the moderate category because of comparatively low epidemiological severity of the baseline with less severe viral hemorrhagic diseases that had high fatality rates. TransmiRisk framework was able to capture this balance by increasing the outbreak component of the risk score but with the moderation of the pathogen-type weighting and transmission features. This case shows the ability of the framework to distinguish between occurrence of the outbreak and biological severity, which makes risk classification proportional and context-dependent.

This analysis shows that TransmiRisk-AI is an adaptive tool to real-time changes in epidemiology.

### 4.2 Performance of the agentic AI system

The agentic system was evaluated for:

- Autonomy
- Fault tolerance
- Timeliness
- Scalability
- Correct detection of risk shifts

#### 4.2.1 Autonomy

The orchestration aspect regulates the end-to-end implementation of the TransmiRisk workflow, which provides a smooth coordination of all the modules of the system. In particular, the orchestrator handles the ingestion of data, hybrid risk scoring, changes in outbreak signals, alert generation, and results snapshoting one by one to enable traceability and audit the outcomes. It is entirely automated with the help of a scheduler that is set to run every specific time period (e.g., every day or every hour) and allows to monitor the situation and re-evaluate the risks. By automating the ingestion-to-alert pipeline, the orchestrator will provide timely updates, less manual efforts, and help track zoonotic events in real-time and make decisions.

#### 4.2.2 Processing time

- Average execution: 0.9–1.4 s
- Scalability: capable of processing datasets up to 10,000 rows due to lightweight architecture

#### 4.2.3 Alert accuracy

Out of 18 high-risk diseases, 100% were correctly flagged by the AlertAgent.

#### 4.2.4 Logging and traceability

LoggerAgent generated detailed logs for all actions, supporting auditability and reproducibility.

### 4.3 Dashboard usability

The decision-support dashboard, which is based on Streamlit, is the interactive visualization layer of the TransmiRisk framework. It offers real-time summary of outbreaks in table form, dynamic heatmap to demonstrate the dynamic pattern of disease emerging, risk distribution chart by category of zoonotic and time-series chart to show fluctuations in the risk with time. Moreover, the dashboard considers agent logs as transparency of the entire system and auto generation of high-risk disease messages to facilitate quick response. The platform converts the multi-dimensional outputs of analytical processes by integrating the complex epidemiological data, hybrid risk scores, and outbreak intelligence into a user-friendly system to turn multi-dimensional outputs into visual information that can be acted upon by the public health decision-makers. This dashboard demonstrates the practical deployment capability of the TransmiRisk framework for real-time zoonotic disease surveillance and decision support.

### 4.4 Visual insights

The TransmiRisk-AI system incorporates an interactive dashboard built with Streamlit for real-time monitoring, risk prediction and transparency. The main elements of the dashboard, showing how it can serve as a decision-support system for health surveillance.

#### 4.4.1 Data upload and agent orchestration

Figure 3 shows data upload and agent orchestration module of the dashboard. It provides an easy to use interface to upload data in a structured format (CSV, Excel) for diseases. The “Run TransmiRisk Agent” button can be used to start the entire data processing pipeline. In the event of the triggering, the orchestrator orchestrates underneath agents for the information ingestion, information preparing, hazard investigation, and alert generation. This module demonstrates system’s learning ability and its ability to automatically execute the data processing pipeline whenever new data is added.

**Figure 3:**
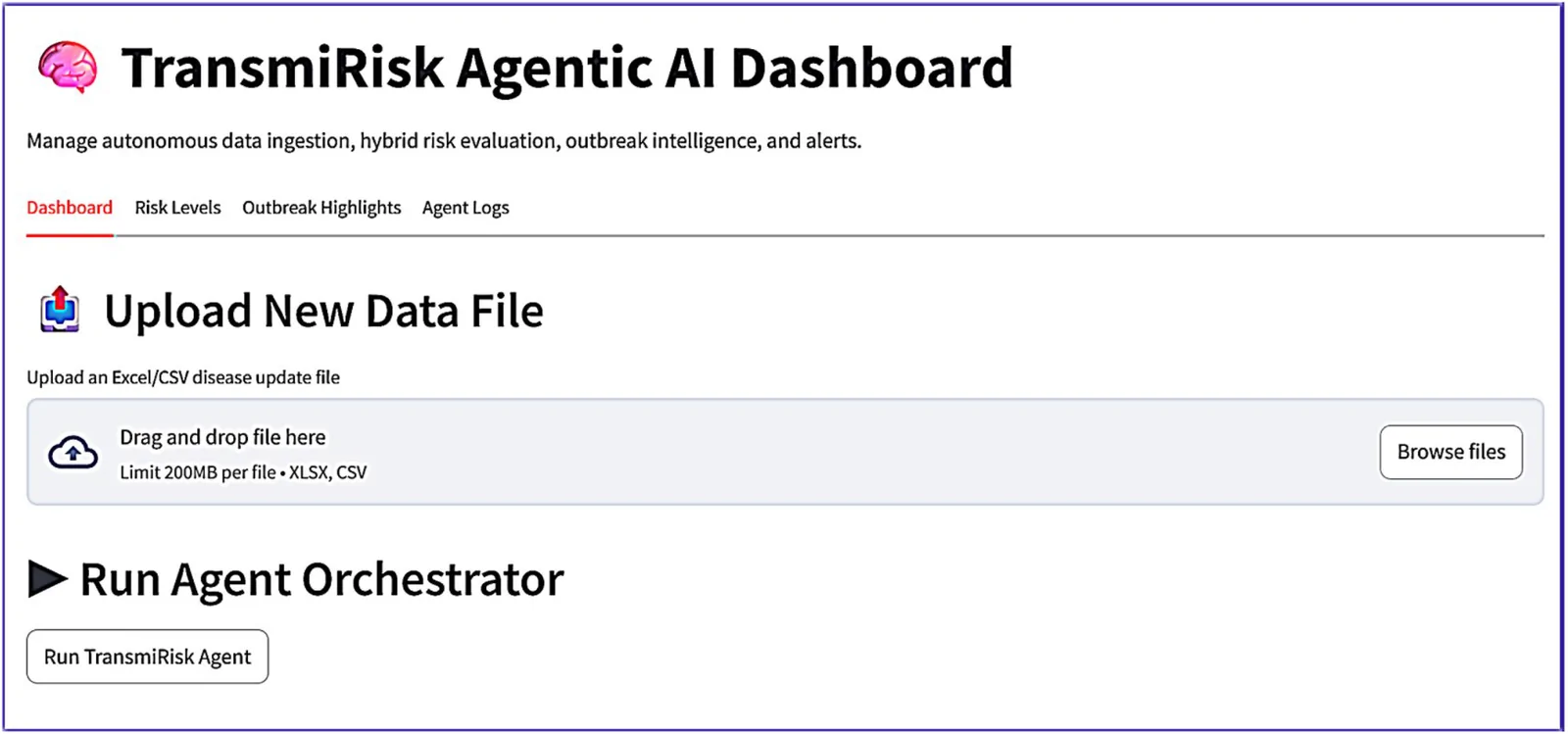
Data upload and orchestration visualization of dashboard.

#### 4.4.2 Risk level assessment and dataset snapshot

Figure 4 shows risk levels module of the dashboard. It offers a current snapshot of the processed data, including outbreak related parameters like outbreak score, cases, deaths and age factors. The master dataset view also includes the latest composite risk scores and risk scores (High, Middle, Low) of each disease. This enables users to track changes in risk for multiple diseases, and ensures outbreak impacts are confirmed in prioritization. Risk tags enable easy identification of high priority diseases.

**Figure 4:**
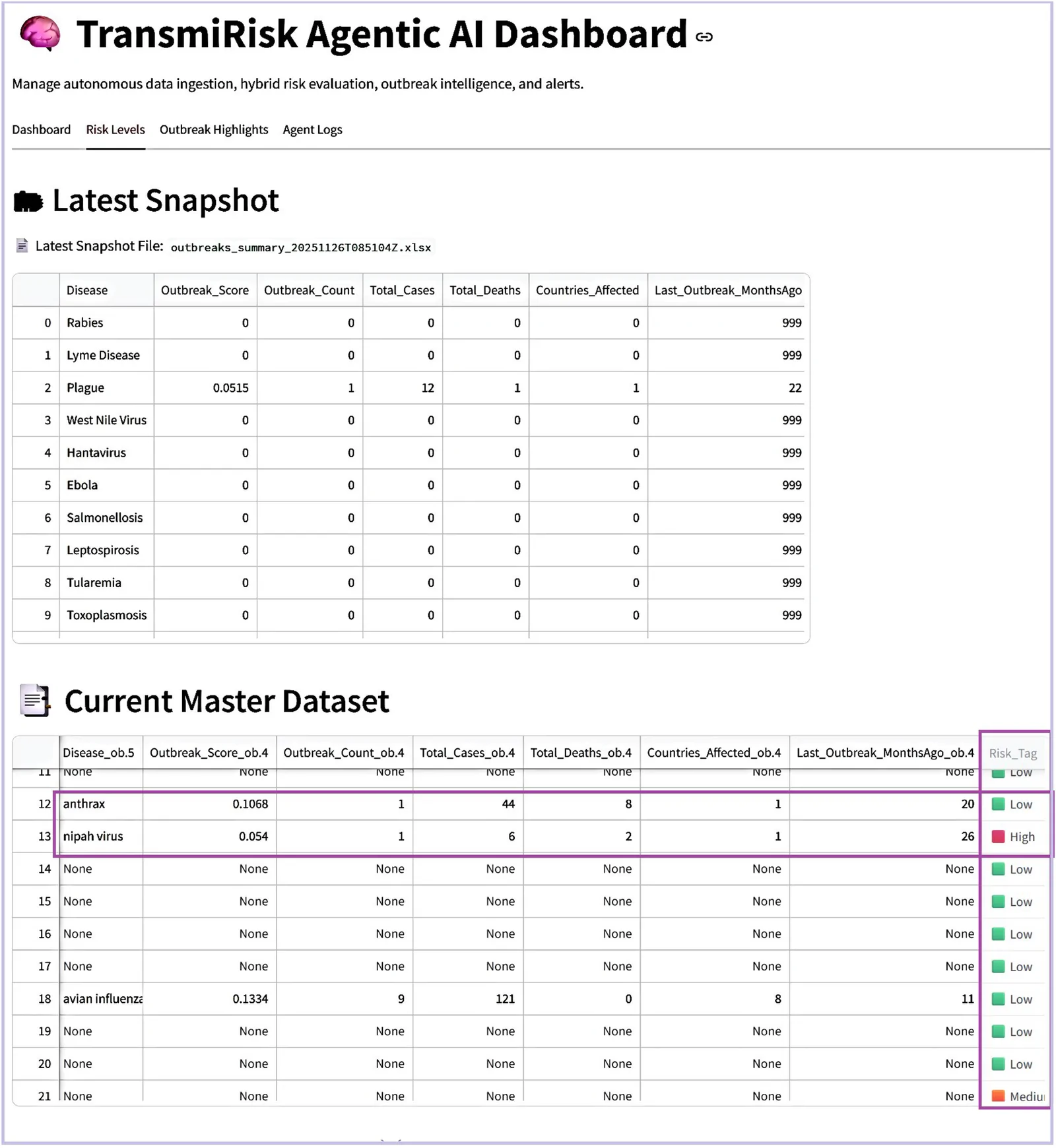
Risk level assessment visualization of dashboard.

#### 4.4.3 Outbreak monitoring and visualization

Figure 5 shows the outbreak monitoring module of the dashboard. This module provides data in real-time for a situational overview. Key features include:

- Important data such as total number of outbreak reports, diseases affected, countries affected.
- Tables of recent outbreak reports with details of cases, deaths and when they occurred.
- Time series of outbreaks to show temporal variability in outbreaks.
- Choropleth maps illustrating the strength of outbreaks by country.
- An integrated picture of recent outbreaks and disease composite risk.

These visuals enable users to understand the temporal and geographic characteristics of zoonotic disease outbreaks, which can assist in decision making based on such evidence.

**Figure 5:**
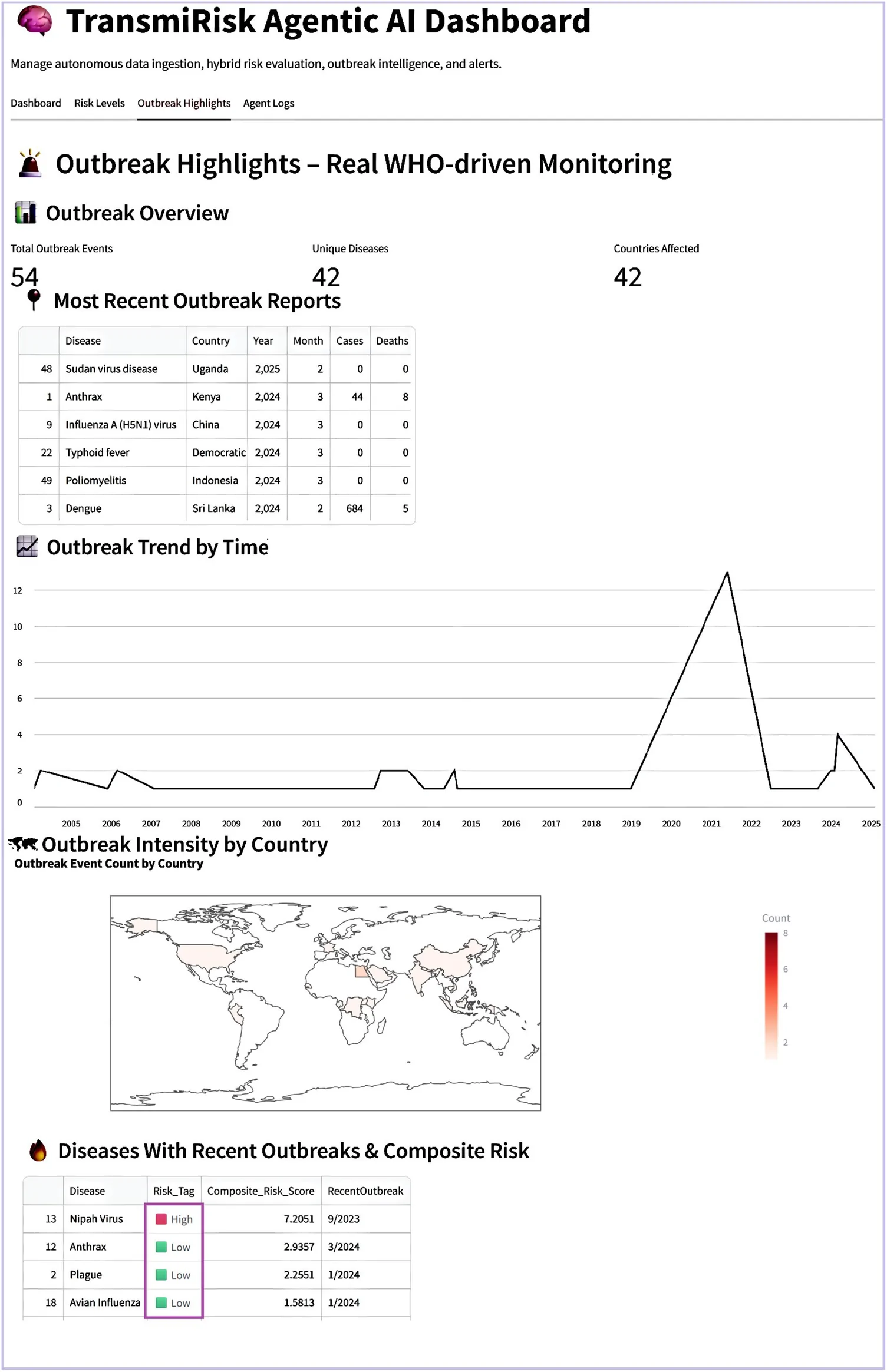
Outbreak monitoring visualization of dashboard.

#### 4.4.4 Agent execution logs and system transparency

Figure 6 shows agent run logs module of the dashboard. It monitors system operations in real-time. These logs record significant events like the start of the orchestrator, data loading, processing status and completion of workflow. This provides complete auditability of actions and improves accountability by enabling users to verify the actions taken by the agentic system. It’s also used for troubleshooting and performance evaluation.

**Figure 6:**
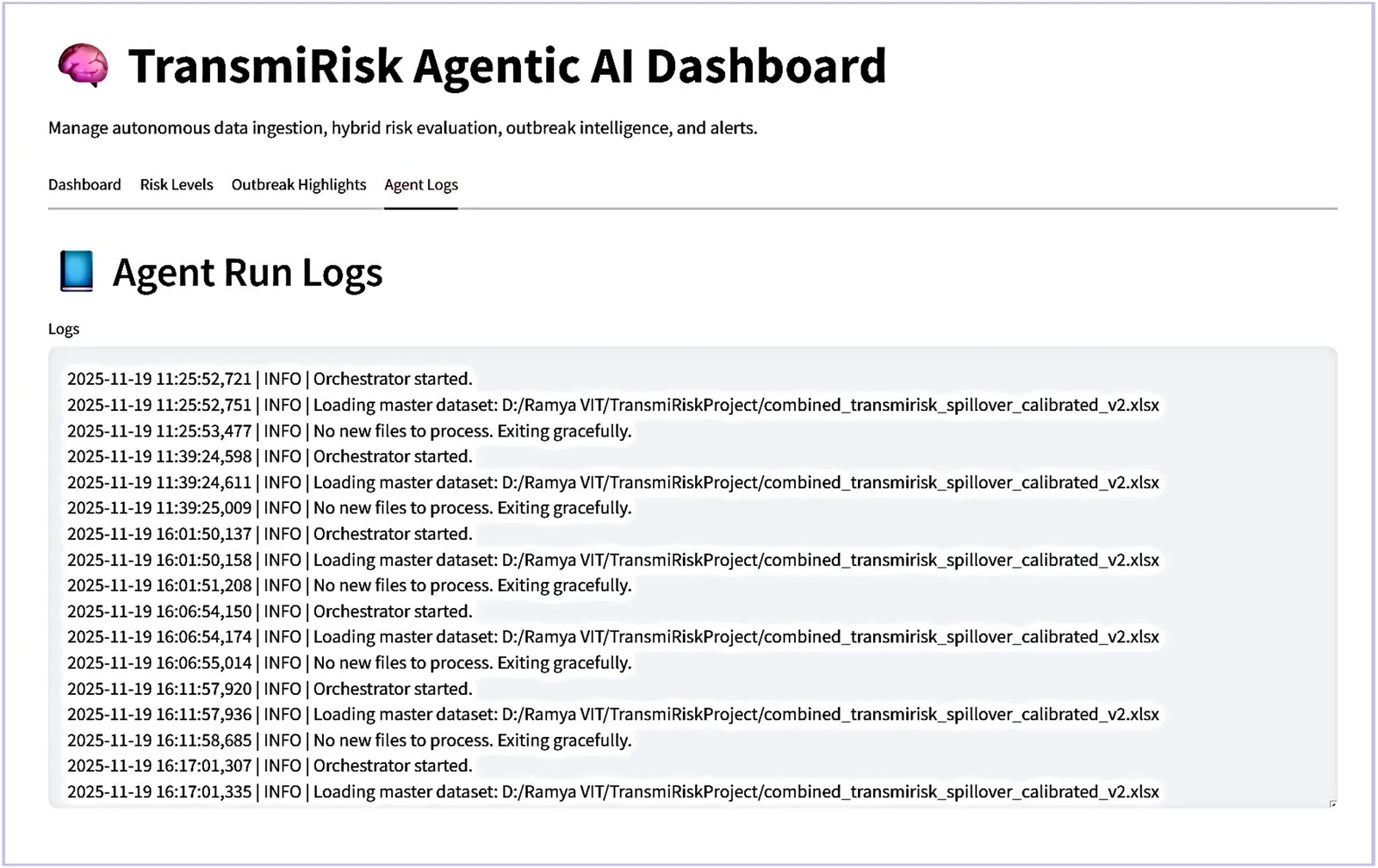
Agent run logs visualization of dashboard.

To conclude, the dashboard is a visualizing tool that is a part of the system and connects analytical results with easy-to-understand information. Integrated with data ingest, risk assessment, outbreak management, and logging, TransmiRisk-AI enhances user experience and enables public health response in real-time.

TransmiRisk-AI demonstrates the following:

- The detection of zoonotic threats with high accuracy across the world.
- Meaningful integration of epidemiological, ecological, and outbreak indicators
- The ability to operate AI agents autonomously.
- Practical and usable for real time public health surveillance.

## 5 Discussion

This study has shown that TransmiRisk-AI offers a viable and scalable solution to real-time surveillance of zoonotic diseases, integrating hybrid risk analytics with an agentic AI architecture. Contrary to the conventional surveillance systems, which work reactively and in a disjointed fashion, the proposed system will provide the opportunity to conduct permanent monitoring, automated decision-making, and prioritization of risks on the fly, eliminating key vulnerabilities in the existing public health infrastructure.

### 5.1 Importance of agentic AI in surveillance

The adoption of the agentic AI paradigm of epidemiological intelligence is one of the main contributions of the work. The multi-agent architecture, which includes data ingestion, risk evaluation, alerting, execution, logging and orchestration agents, allows end-to-end automation of the surveillance pipeline. This design will greatly minimize manual intervention, uniformity in data processing, and prompt identification of the risks that occur. The orchestrator is important in ensuring coordination of workflow and thus the system is both robust towards scheduled and event-driven execution.

The fact that the agents are able to act independently and that they are traceable through logging mechanisms also increases efficiency and transparency in operations. This is especially so in health care settings, especially in the public where decisions and accountability are crucial.

### 5.2 Adaptation of dynamic risk and real-time responsiveness

One of the advantages of TransmiRisk-AI is its ability to respond to real-time outbreak indicators. The system dynamically re-calibrates risk scores of diseases based on recency, frequency and severity of reported events, by incorporating outbreak intelligence into the risk evaluation pipeline. This allows an adaptive prioritization to happen, in which the diseases could move along risk categories as more data is made available.

The fact that the case study analyses, including the risk escalation in Crimean–Congo Hemorrhagic Fever and the risk changes increment in the Nipah virus outbreaks, reflect the ability of the system to capture the temporal dynamics is successful. This dynamic flexibility is a huge improvement on the old-fashioned prioritization systems that are usually not responsive to the fast-changing epidemiological realities.

### 5.3 System performance and practical utility

The experimental analysis shows that the system has an efficient execution with a low processing time and an excellent alert accuracy. The fact that updates can be processed in seconds points out its appropriateness in real-time deployment. Another aspect, the precise determination of high-risk diseases, and the correct detection of the transitions between risk categories confirm the quality of the underlying workflow.

In terms of usability, the addition of a dashboard based on Streamlit makes it easier to access by the stakeholders of the public health. The platform can enable informed decision-making without the need to possess advanced technical skills by converting complex analytical results into easily understandable visualizations, like risk categories, outbreak trends, and system logs.

### 5.4 Comparison with existing systems

Current outbreak intelligence systems are mainly centered around reporting and early warning indicators, in contrast to the built-in risk quantification and automation. On the same note, a lot of AI-based models lack multi-disease prioritization and real-time flexibility, being disease-specific. TransmiRisk-AI overcomes these shortcomings by integrating multi-source data, hybrid risk scores, and agent-based automation in a single framework.

Also, whereas in the past, non-explainable and non-transparent systems were common, logging mechanisms and interpretable risk outputs in TransmiRisk-AI raise the level of trust and usability among decision-makers. This makes the system a transition between data-driven analytics and usable intelligence on public health.

### 5.5 Generalizability

Generalizability to Other Endemic Diseases: The proposed Agentic AI architecture is an extensible surveillance framework that is not endemic disease specific. The existing TransmiRisk database comprises 124 zoonotic diseases, including the Dengue virus (DENV), which can be evaluated in the existing framework of risk assessment. The modular design of the agent architecture allows the application of the same workflow to other diseases by adding disease-specific epidemiological, ecological, and outbreak information. A possible extension, Chagas disease, will be added when the needed standardized information is available. These extensions will have to be independently validated to ensure that the disease-specific risk characteristics and surveillance signals are represented.

### 5.6 Limitations

Although it has some strong points, there are a number of limitations to be considered. Real-time data on outbreaks may vary in quality and availability between regions and diseases and determines the efficiency of the system. Late or missing reporting may affect the accuracy of risk estimation. Also, although the agentic architecture offers automation, it is not yet based on adaptive learning or self-optimization, and is instead based on predefined workflows.

The ongoing implementation is also not based on the complete integration of other influential factors, such as climate variability, mobility patterns, or genomic data, which are known to influence zoonotic transmission dynamics. The addition of these dimensions may also improve predictive potential and situational awareness.

The diagnostic approach is a rule-based assessment that is deterministic and reproducible; however, the scoring criteria and thresholds have been methodological choices that have not been statistically optimized to an independent gold standard outcome. As a result, the categories obtained represent relative risk characterisation levels, not “calibrated” probabilities of zoonosis transmission. Future studies will assess for other weights and thresholds as well as test the framework with independent epidemiological and outbreak data sets.

### 5.7 Implications to public health

TransmiRisk-AI has profound implications for contemporary public health surveillance. The system facilitates proactive intervention measures and effective allocation of resources, as it allows the early detection of diseases with high risks and continuous monitoring of risks. The automated alerting system will keep people aware of critical risk signals in real time, minimizing lag in response time.

In addition, the framework is aligned with the One Health approach as it helps to integrate different data sources and cross-sectoral collaboration. Its modularity and scalability to regional, national, or global levels make it an option to implement a pathway towards intelligent, data-driven surveillance ecosystems.

In general, TransmiRisk-AI is a significant step towards autonomous, explainable, and real-time zoonotic disease monitoring, which is a significant issue in the modern public health system.

## 6 Conclusion

This article introduces TransmiRisk-AI, an agent-based artificial intelligence system that is intended to be used for real-time surveillance and risk monitoring of zoonotic diseases in an autonomous manner. The proposed system overcomes key shortcomings of conventional surveillance methods such as the absence of automation, slow reaction, and minimal flexibility to changing outbreaks by combining a hybrid risk scoring model with a coordinated multi-agent architecture.

The agentic design, consisting of data ingestion, risk evaluation, alerting, execution and orchestration agents, enables the ongoing data processing, dynamic risk recalculation and automated dissemination of alerts in real time. Real-time outbreak intelligence makes the system more responsive to changes in disease risk profiles as new events appear and enable it to detect and reflect them. Moreover, the interactive dashboard allows the intuitively visual representation of risk categories, outbreak trends, and system logs, enhancing situational awareness of the public health stakeholders.

Experimental findings show that TransmiRisk-AI is an efficient model in execution time, scalability, and accuracy of alerting, and it can effectively model the outbreak-driven changes in zoonotic risk. The case studies also confirm that the system is able to adjust to real-world epidemiological shifts and offer actionable information to make decisions.

Altogether, TransmiRisk-AI adds a scalable, explainable, and autonomous surveillance technology that covers the gap between operational public health intelligence and risk modeling with data. The framework shows how agentic AI can turn traditional surveillance systems into proactive, constantly learning ecosystems to help make timely and informed interventions in zoonotic disease control.

## 7 Future work

Although TransmiRisk-AI is a promising model that can be applied in real-time to monitor the risks of zoonosis, it can be improved in a number of aspects to enhance its predictive power, scalability, and application to the real world. The future research will involve the incorporation of climate and environmental forces like temperature, rainfall, and ENSO patterns with remote sensing data, as well as the ecological features and suitability of a habitat by employing the models of both the ecological features and suitability of a habitat to support the climate-sensitive risk predictions. Also, genomic surveillance information provided by such platforms as GISAID and Nextstrain may be used to identify new variants and mutations that are linked to high transmissibility at an early stage. The addition of mobility, trade and human behavior data, including air travel, migration patterns and livestock movement will make prediction of outbreaks and assessments of risks across borders possible through networks.

Future developments will look into machine-learned predictive modeling, such as spatiotemporal deep learning, LSTM forecasting, graph neural network spillover pathways, and reinforcement learning adaptive surveillance strategies. The agentic AI paradigm can be extended to goal-oriented autonomous systems which can include multi-agent reasoning, natural language outbreak summarization, and automatic data discovery. In addition, it will increase the effectiveness of its operations by implementing TransmiRisk-AI as a scalable cloud-based system of national and international surveillance systems, including One Health initiatives and global organizations. By including human-in-the-loop systems and policy-based interfaces, further expert validation, decision-making, and effective resource allocation in the health systems will be enhanced.

## Code availability statement

The source code for the TransmiRisk-AI agentic surveillance framework is maintained by the authors. The implementation can be provided by the authors upon reasonable request to support reproducibility and further research.

## Data Availability

The raw data supporting the conclusions of this article will be made available by the authors, without undue reservation.
